# Using a stated preference discrete choice experiment to assess societal value from the perspective of patients with rare diseases in Italy

**DOI:** 10.1186/s13023-019-1126-1

**Published:** 2019-06-26

**Authors:** Julio López-Bastida, Juan Manuel Ramos-Goñi, Isaac Aranda-Reneo, Domenica Taruscio, Armando Magrelli, Panos Kanavos

**Affiliations:** 1 Faculty of Health Science, University of Castilla-La Mancha, Talavera de la Reina, Toledo Spain; 2 Axentiva Solutions, Tacoronte, Spain; 3 Faculty of Social Science, University of Castilla-La Mancha, Talavera de la Reina, Toledo Spain; 4 Istituto Superiore di Sanitá, Rome, Italy; 5 Faculty of Pharmacy, University of Rome, Rome, Italy; 6 Department of Social Policy and Medical Technology Research Group, London School of Economics and Political Science, London, UK

**Keywords:** Discrete choice experiment, Cystic fibrosis, Haemophilia, Decision making, Rare disease, Orphan drugs, Italy

## Abstract

**Background:**

Decision makers have huge problems when attempting to attribute social value to the improvements achieved by new drugs, especially when considering the use of orphan drugs for rare diseases. We present the results of a pilot study aimed to investigate patient preferences regarding public funding for drugs used to treat rare diseases.

**Methods:**

An online questionnaire was used as a discrete choice experiment (DCE) survey to explore the preferences of patients with cystic fibrosis and haemophilia in Italy. The questionnaire focused on relevant issues that were defined in a review of the literature. A conditional logistic model showed preferences for specific attributes.

**Results:**

A total of 54 questionnaires (20% response rate) were completed. The issues that received the greatest attention were improvement in health, treatment cost and value for money. However, disease severity and the availability of other treatments were important social values that could not be ignored.

**Conclusions:**

The findings presented here provide evidence as to what patients with cystic fibrosis or haemophilia think are the most important considerations on which to base decisions in health technology scenarios, and regarding the priorities for funding.

## Background

Since the price and effectiveness of therapies for rare diseases often suggest they are unlikely to provide value for money, in some countries it may be necessary to use additional criteria when assessing reimbursement, including disease severity, improvement in health and the availability of alternative treatments [1]. Drug pricing is a national competence exercised by the Italian medicines agency (AIFA). Orphan drug regulations follow the same indications of all other medicines. The price at which a medication will eventually be reimbursed by the National Healthcare System (NHS) is the result of a negotiation between the company requesting the medicine market access in Italy and AIFA. Indeed, in such circumstances multi-criteria decision analysis (MCDA) represents a useful aid to decision-making. In MCDA, the relative importance and the influence of each criterion on the final decision is defined [2]. Although MCDA are not commonly employed in health technology assessment, they are important aids to decision-making when establishing health priorities and as such, they are being encountered more frequently [3, 4]. Indeed, when multiple conflicting criteria, goals or objectives have to be taken into account, MCDA are increasingly recognised as a valuable aid when faced with complex decisions. As a result, one MCDA technique has become increasingly popular, the Discrete Choice Experiment (DCE). This approach can provide stated-preference information, indicate whether particular attributes are predictors of choice in different scenarios, as well as assess the relative importance of the attributes used to describe the alternatives in choice sets [5]. There is evidence that a DCE approach may be suitable to establish general preferences and to guide priorities in healthcare provisions [5, 6]. Indeed, establishing a decision-making framework that extends beyond Cost-QALY approach and that takes into account additional criteria may well represent a fairer system in the context of rare diseases.

In the light of the above, a pilot study has been carried out to determine the preferences of cystic fibrosis and haemophilia patients in Italian registries regarding healthcare interventions, and particularly in reference to generic social value judgments. The study uses a DCE framework to consider the patient’s preferences and it specifically explores how patients may weigh up competing distributive preferences in a priority-setting context.

## Methods

A DCE was carried out in accordance with previously used methods [5]. More details about the design are provided elsewhere [7]. The study followed the approach recommended by Street and Burgess [8].

### Attributes and levels

A systematic review of the empirical literature on distributive preferences was carried out to inform the attribute selection [9]. This literature review aimed to identify the specific attributes suitable to design a DCE for rare diseases that would help develop and validate a framework to support decision-making. Attributes were selected based on the frequency of their use in rare disease-related literature. The following attributes were chosen: improvement in health; treatment cost; side effects; waiting time; disease severity; availability of other treatments; and value for money. This list was discussed with methodological experts and healthcare decision-makers to confirm the validity of the criteria selected. Finally, we defined eight attributes; seven identified through the literature review and one more recommended by the expert panel: beginning of life (i.e. patients younger than 10 years of age).

The relative importance of each criterion was assessed in a pre-pilot study carried out to achieve an attribute ranking, which helped define the best way to present the information. Based on a descriptive analysis of the results obtained and another round of discussion with the expert panel, the final attribute selection was made. Then, a formal pilot study was carried out using an interview between a random sample of the expert panel, and most of the respondents indicated that they could understand and complete the questions. Indeed, the respondents provided positive comments regarding the level of understanding and their commitment. The selection of the attribute levels took into account two main criteria: first, to preserve the number of evidence-based attributes given their importance in the decision-making process; and second, to ensure the feasibility of the experimental design. In Table 1, the attributes selected, and the specific levels are described.

**Table 1:** Discrete choice experiment attributes and levels

Attribute	Levels
Disease Severity	Moderate
	Severe
Health Improvement	Large
	Moderate
	Small
	Very small
Waiting times	Short
	Moderate
	Long
Availability of other treatments	Yes
	No
Side effects	Few
	Moderate
	Many
Value for money	Very good
	Fairly good
	Fairly poor
	Very poor
Beginning of life	Yes
	No
Treatment Cost	Zero
	Low
	Moderate
	High

Source: a full explanation of attributes and levels are provided on Lopez-Bastida, et al. (2018) [7]

### Experimental design

An orthogonal, main-effects design was adopted that included 36 pairs of scenarios distributed into two equal sized blocks. Each scenario described a combination of attributes and levels, with known efficiency, and it used fold-over copies to create the necessary subsequent choices. The design assumed that interactions among the attributes were insignificant in all the two-way and higher-order interactions [5]. A blocking approach was selected to limit respondent burden, and the design was balanced evenly across the blocks. Respondents were asked to make a series of choices involving two alternative healthcare scenarios (paired comparisons).

The design used had level balance and was orthogonal, minimising multicollinearity. All of the scenarios used were checked for plausibility, i.e. the potential that health technologies would fit the scenarios. The choices were blocked into two sets of 18 choices to avoid the respondent burden, for two versions of the questionnaire. The blocking into two sets of questions was undertaken using an additional attribute column from the factorial design employed, to ensure orthogonality over the choice set.

### Sample/data collection

**Fig. 1:**
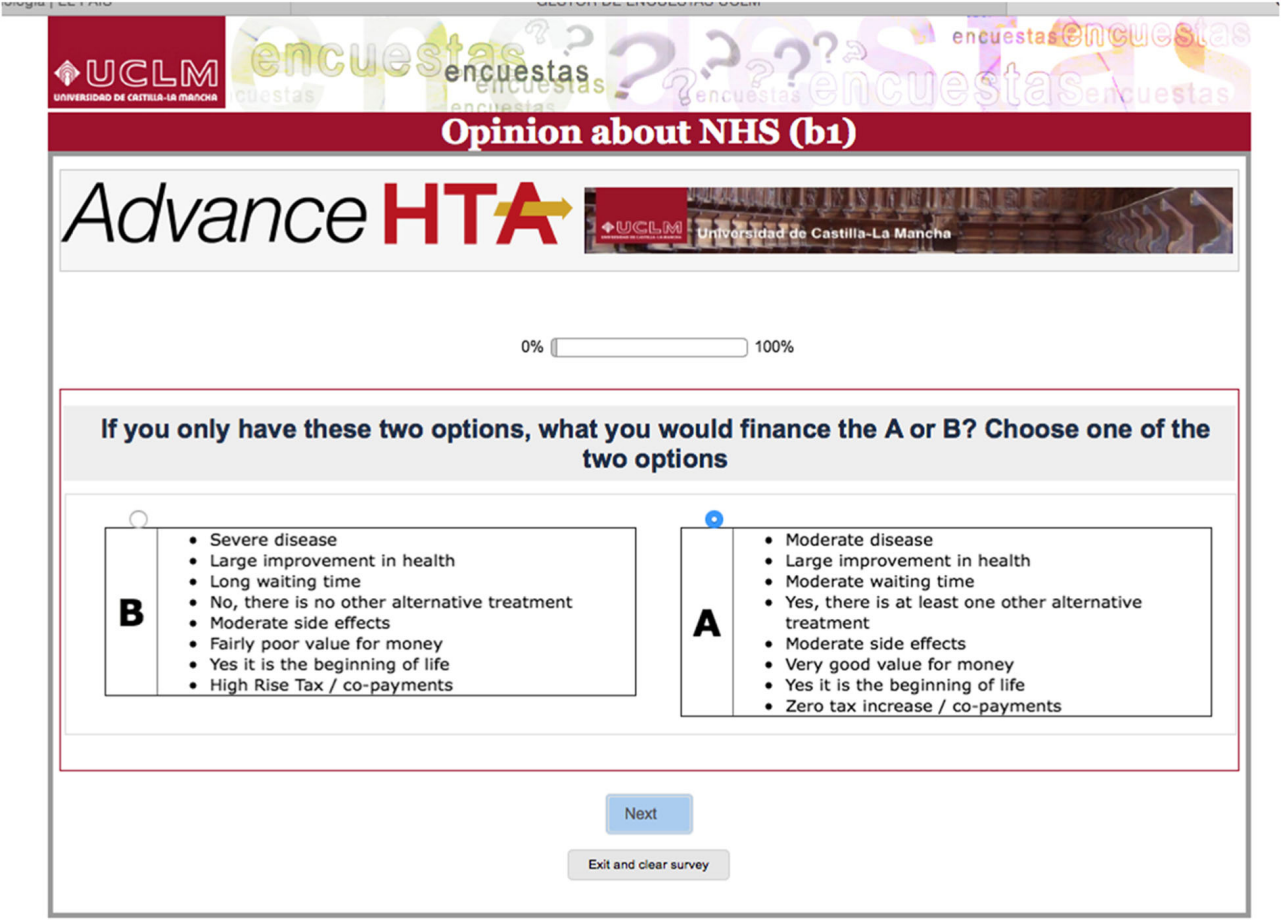
Example of a survey question

Researchers participating in the ADVANCE-HTA Project (Italy) contacted cystic fibrosis or haemophilia patients from the registries of existing Italian Haemophilia and Cystic Fibrosis Associations. The Italian registry of CF counts with 5362 patients and 120 were invited to complete the questionnaire. The Italian federation of haemophilia (FEDEMO), which includes 32 Italian local association counts with 9000 haemophilia patients. The haemophilia association invited 150 patients to answer the questionnaire. The patients were invited to participate directly by their patient’s associations and they were asked to complete an online questionnaire. Data were also collected during control visits to hospitals (in particular for Cystic Fibrosis patients). The attributes and levels were presented as features of health technologies, and the patients were asked to put themselves in the context of a health service decision-maker faced with difficult decisions in a priority setting. One of the two alternative scenarios in each choice set had to be selected since the decision-maker was unable to fund all of the health technologies (Fig. 1 shows an example of one question in the survey).

There is limited consensus on sample size calculations for discrete choice studies, and there are no well-designed, practical rules to guide the analyst [10, 11]. Thus, we tried to maximise the sample size, obtaining a minimum of 20 responses per block to obtain sufficient variance in the estimated choice probabilities.

### Data analysis

A descriptive analysis was used to present the background characteristics of the respondents. Means and standard deviation were used for continuous variables, and proportions for dichotomous variables. The responses variables were categorised in two values, ‘1’ represented the option being chosen and ‘0’ the one not chosen. Following Mcfaden’s framework based on the random utility theory, preferences estimates rest on the assumption that when participants chose scenario A over scenario B, scenario A gave them a higher utility. Accordingly, a conditional logistic model can be applied, and the coefficients of the model represent the relative weights of each level of each attribute, allowing the interpolation or extrapolation of the utilities not observed within the population [11, 12]. In other words, the coefficients of the model can be interpreted to define the relative importance that the sample gave to the movement of any given attribute from the reference level to a different level. Given the coding of the levels by attribute (a = reference case, b = 1, c = 2 and so on), positive and negative coefficients could arise. For example, for the attribute related to the availability of alternative treatments, the reference case (a) is “Yes”, so that the coefficient of level b will show the change in the utility when moving from “Yes” to “No”. In this case, we expect a positive coefficient, and if there is no other existing treatment, the chance of being funded would be higher. However, for the attribute related to disease onset at the beginning of life, the opposite is the case, and we would expect a negative coefficient. Finally, an exclusion criterion was applied for respondents who took 200 s or less to complete the survey in order to eliminate random (non-meaningful) responses. All statistical analyses were performed using STATA MP [13].

## Results

A total of 54 questionnaires were completed (20% response rate), and of these, eight were excluded from the analysis as they failed to comply with the exclusion criteria described in the method section. Thus, the valid sample comprised of 46 patients, the main characteristics of which are shown in Table 2. The sample sizes by block surpassed our expectations (> 20 respondents), although the exclusion criteria removed six respondents from block 2 and two respondents from block 1 (Table 2).

**Table 2:** Characteristics of participants in the DCE

	Before exclusion criteria (*n* = 54)	After exclusion criteria (*n* = 46)
Age, mean (SD)	38.98 (10.27)	38.36 (9.24)
Household members, mean (SD)	2.72 (1.28)	2.65 (1.22)
Self-reported health status, %		
Good	66.67	71.74
Average	7.41	4.35
Poor	25.93	23.91
Patients answering block 1, n (%)	25 (46.29)	23 (50)
Patients answering block 2, n (%)	29 (53.7)	23 (50)
Interview duration^a^, mean (SD)	415.1 (301.45)	460.21 (304.42)
Task time^a^, mean (SD)	21.21 (50.48)	23.66 (54.3)
Were the questions easy to understand? (%)		
Agree	40.7	47.8
Mildly agree	31.5	32.6
Indifferent	11.1	10.9
Mildly disagree	11.1	6.5
Disagree	5.6	2.2

^a^Time in seconds

The observed choice probabilities ranged from 30.43 to 69.5%, which means that different decisions will be made for an individual scenario depending on who makes the decision, with no clear preferences among the patients. The coefficients in Table 3 reflect the partial worth utilities associated with changes in each of the attribute levels (compared to the reference case). For example, a health technology scenario with a large improvement in health has a higher utility/preference than scenarios with a small or moderate improvement in health, all else being equal. Each logit model coefficient showed similar patterns, with many coefficients close to 0 due to the response of the model to the observed probabilities. The closer the probabilities are to 50%, the smaller the expected distance between the two options in the DCE for that utility. Accordingly, the improvements in health, treatment cost and value for money were the attributes that received the most attention from patients, while disease severity and the availability of other treatments were less important to these patients (Table 3). The model fits the observed probabilities well (i.e., those included in the DCE design), and the models do not show probability inversions. The range of the probabilities, showing the uncertainty in the estimates.

**Table 3:** Logit model coefficients

Attribute	Level	Model framework	
		Logit coefficients	Confidence interval (95%)
Severity of the disease (reference = moderate)	Severe disease	0.005	−0.309-0.318
Improvement in health (reference = large)	Moderate	−0.425	− 0.949-0.098
	Small	−0.369	− 0.925-0.186
	Very small	−0.070	−0.353-0.213
Waiting times (reference = short)	Moderate	−0.102	−0.383-0.18
	Long	0.020	−0.256-0.296
Availability of other treatment (reference = yes)	No	−0.008	−0.303-0.286
Side effects (reference = few)	Moderate	−0.079	−0.37-0.213
	Many	−0.002	−0.286-0.282
Value for money (reference = very good)	Fairly good	−0.285	−0.753-0.182
	Fairly poor	−0.042	0–0
	Very poor	−0.301	−0.629-0.028
Beginning of life (refeence = yes)	No	0.158	−0.085-0.401
Cost of treatment (measured by tax increase / copayments) (reference = none)	Low	0.396	−0.13-0.922
	Moderate	0.361	−0.09-0.811
	High	0.112	−0.241-0.465

None of this coefficient was statistically significant

## Discussion

When making priority-setting decisions, policymakers are often faced with difficult choices between options that can each be regarded as potentially beneficial. However, a range of social values will influence policy decisions, and when there are trade-offs between such social values, or equity arguments (objectives), the judgment must be made as to which is the best decision.

In recent years, several frameworks have been prepared in which an MCDA guides reimbursement decision-making for orphan drugs [13–16]. Here, a pilot MCDA study was used to establish a framework of weighted attributes that could serve to attribute value to orphan medicinal products. From the results obtained, it is clear that this type of approach could be developed further to aid health technology assessment bodies and payers.

No previous studies focus on cystic fibrosis or haemophilia have been found. Thus, the findings presented here provide evidence as to how patients with cystic fibrosis and haemophilia think that decisions should be made in Italy regarding which health technology (orphan drugs) scenarios are worthier of receiving funds. Improvements in health, treatment cost and value for money are the attributes that received the most significant attention of patients with these rare diseases, while disease severity and the availability of other treatments were less important to these patients. There may be a link that both diseases are genetic/inherited that may influence the results.

The most important attributes for this group of patients have similarities with the group of decision-makers that we have tested in a similar study [7]. In addition, the results in this study are in line with earlier work from England, where face-to-face interviews were used on a sample from the general population, and where improvement in health and value for money were the attributes that provided the strongest indication of social value (preference) [5].

This is valid information that can be used to design future Bayesian-based DCE designs. Indeed, the information gathered here could serve to inform an efficient Bayesian design in order to obtain a general algorithm that would help make a uniform decision across the healthcare system.

This study has some limitations, reflecting its explorative nature. The experimental design used was not complex, with a small factorial design used, and the results presented here are based on a simple analytical framework, using a conditional logistic model. Both of these factors are deliberate to ensure that the findings are policy-relevant and that they are presented in a policy friendly manner, although they do represent potential limitations. As an exploratory study, we sought to keep the experimental design straightforward, albeit in the knowledge that these preliminary findings could be used to inform more detailed future study designs. The attributes considered covered issues that were expected to be very common in priority-setting dilemmas, such as technology assessment. However, some patients may think that their specific health problems are not covered by the criteria used. Of course, other attributes could be introduced to make the scenarios more context specific. Indeed, a further limitation of the study is the absence of objective data on the seriousness of the response for the sample due to the use of an internet-based strategy. Given the time required to complete the survey, in some cases, we are less confident about the quality of the responses. Nevertheless, we did try to correct for this possible bias by excluding the very short surveys.

It is also possible that the sample provides a good representation of the rare disease patient in Italy and while it is relatively small, it does appear to provide some indication of the extent to which the findings can be generalised. Indeed, the feedback from interviewers from the pilot study (expert panel) and the main survey (patients) was that the respondents were keen to participate, they seemed engaged in the survey, and generally, they had few problems completing the survey.

As with most empirical studies of this type, the study is also open to some level of criticism regarding the presentation, framing and the contextual approach adopted. However, it does appear that the results are useful and indicative of what may be possible if more comprehensive research initiatives of this nature were carried out. Other ways of administering surveys should also be examined in such explorative study, such as face to face interviews, which may shed light on how respondents think when they are completing the task. More importantly, such approaches may help gain engagement with the research through a good structure personal presentation. Thus, an increase in interviewer-administered DCE would represent a clear development, and accordingly, future work should explore the inclusion of interactions in the design and analysis stages of a DCE. On the other hand, it has been recently published a high sample size DCE study where the participants who completed the DCE also using an online tool. The authors concluded that including overlap when presenting the choices or using different colours for each level could make more accessible for participants to identify the differences between levels and moreover it seems that combining colour editing levels plus overlap reduced the dropout rate [17]. In our study, the dropout rate and the participants who stated the DCE “was easily understood” were very low. We are in line with Jonker et al. and we think that using overlap or maybe colour editing our comprehension-related problem of some participants who took the DCE could be solved.

## Conclusions

This study adds to the relatively sparse literature on the use of DCE methods to explore preferences about health service funding. The information presented here could be useful for future research designs following similar approaches. Nested designs using partial profile presentation should be explored to gain in both the understanding of individual preference and in the clarity of the questions presented. The feasibility and acceptability of the DCE approach to determine patient preferences over priority-setting scenarios for healthcare provision has several limitations, mostly due to the heterogeneity in the preferences stated. However, several considerations should be made in order to design an appropriate experiment and in particular, we highlight the need to use “face to face interviews instead of online surveys”. The results of a DCE could be an additional HTA policy tool in the assessment of the price and reimbursement dossiers for orphan drugs in Italy.

## Data Availability

The data that support the findings of this study are available from the corresponding author upon reasonable request.

## Abbreviations

ADVANCE-HTA: Advancing and strengthening the methodological tools and practices relating to the application and implementation of Health Technology Assessment
DCE: Discrete choice experiment
MCDA: Multi-criteria decision analysis
QALY: Quality-adjusted life year
